# A Comparative Study of Magnesium Sulfate, Lignocaine, and Propofol for Attenuating Hemodynamic Response During Functional Endoscopic Sinus Surgery Under General Anaesthesia: A Prospective Randomized Trial

**DOI:** 10.4274/TJAR.2024.241573

**Published:** 2024-10-30

**Authors:** Malipeddi Vamshidhar, Vandana Pakhare, Sunanda Gooty, Ananya Nanda, Ramachandran Gopinath, K. Dilip Kumar, Vyshnavi R

**Affiliations:** 1 ESIC Medical College & Hospital, Sanathnagar, Hyderabad, Department of Anaesthesia and Intensive Care, Hyderabad, India

**Keywords:** Propofol, lignocaine, hypotension, hemodynamic response, magnesium sulfate

## Abstract

**Objective:**

This study functional endoscopic sinus surgery (FESS) is a surgical procedure requiring minimal bleeding to optimize the surgical field. This study aimed to evaluate the effectiveness of magnesium sulfate, lignocaine, and propofol in attenuating hemodynamic response. The primary objective of this study was to compare the efficacy of these agents in reducing hemodynamic response. The secondary objectives included assessing the quality of the surgical field, recovery time, and total neuromuscular dose.

**Methods:**

We randomly allocated 105 patients scheduled for FESS into three groups: lignocaine, propofol, and magnesium sulfate. Heart rate and mean arterial pressure were recorded every 5 min for the first 30 min, followed by measurements every 10 min at the end of the procedure. Moreover, recovery time, total neuromuscular blocking dose, and surgical field score were noted upon completion of the procedure. Statistical analysis was conducted using the number cruncher statistical systems version 9.0.8 software.

**Results:**

All three groups showed comparable hemodynamic response and surgical field scores. The recovery time was notably longer in the magnesium sulfate group [10.94 min (2.45)] than in the lignocaine [4.37 min (1.03)] [95% confidence interval (CI) -7.32, -5.83;* P*=0.000] and propofol groups [4.60 min (0.60)] (95% CI 5.60, 7.095; *P*=0.000). Moreover, the total neuromuscular blocking agent used was significantly lower in the magnesium sulfate group [5.89 mg (0.47)] than in the lignocaine [6.26 mg (0.56)] (95% CI 0.66, 0.03; *P*=0.035).

**Conclusion:**

Propofol, magnesium sulfate, and lignocaine exerted equal efficacy in attenuating hemodynamic responses during surgery and ensuring a satisfactory surgical field. However, magnesium sulfate led to significantly longer recovery timescompared withpropofol and lignocaine. In addition, magnesium sulfate required a significantly lower total dose of neuromuscular blocking agentsthan lignocaine.

**Main Points**

• In our study we aimed to compare the effects of MgSO4, lignocaine, and propofol on attenuating hemodynamic response during functional endoscopic sinus surgery.

• Our primary aim was to compare the hemodynamic attenuation response among the study drugs.

• Our secondary aims were to compare the quality of the surgical field, recovery time, and total neuromuscular dose requirement.

• We concluded that propofol, MgSO4, and lignocaine were equally effective in attenuating the hemodynamic response to surgery and achieving a satisfactory surgical field.

• However, the recovery time was significantly longer with MgSO4 than with propofol and lignocaine.

• The total neuromuscular blocking agent dose was significantly lower with MgSO4 than with lignocaine.

## Introduction

Functional endoscopic sinus surgery (FESS) is a minimally invasive technique aimed at enlarging the nasal drainage pathways of the paranasal sinuses and improving sinus ventilation. This procedure is generally used to treat chronic rhinosinusitis that is unresponsive to drugs, nasal polyps, and certain cancers and to decompress the optic nerve in Graves’ ophthalmopathy. The sinonasal mucosa is highly sensitive and vascular; even minor bleeding can impair surgical field visibility, prolong the procedure, and reduce the quality of the intervention.^1^ This may necessitate blood transfusion and increase the risk of complications like optic nerve injury, orbital cellulitis, meningitis, and rhino-oral fistulas.

An important modality for minimizing this bleeding is the attenuation of the hemodynamic response associated with endoscopic maneuvering. This can be achieved with topical vasoconstrictors, local anaesthesia, or controlled hypotension with drugs like propofol, magnesium sulfate, nitroglycerin, lignocaine, dexmedetomidine, and esmolol.^2, 3, 4^ However, these methods present significant challenges, including drug resistance, tachyphylaxis, cyanide toxicity, and delayed recovery.^3^ Specifically, magnesium sulfate, lignocaine, and propofol are easily available, cost-effective, and have a high safety margin. Although these drugs have been evaluated in previous studies, they have not been compared for their efficacy in reducing hemodynamic responses to FESS. In our study, we aimed to compare magnesium sulfate, lignocaine, and propofol for their ability to attenuate hemodynamic response, improve the quality of the surgical field, reduce recovery time, and decrease the total neuromuscular dose requirement during FESS.

## Methods

After receiving approval from the Institutional Ethics Committee of Employees’ State Insurance Corporation Medical College Hospital & Super Speciality Hospital (approval no.: ESICMC/SNR/IEC-DNB/S002/08/2019, date: 29.08.2019) and registration with the Clinical Trial Registry India (CTRI/2020/06/025648, www.ctri.nic.in), this prospective randomized trial was conducted over a period of one year, from September 1, 2020, to August 31, 2021, in compliance with the Declaration of Helsinki of 1975, as revised in 2013. All eligible participants were informed about the study, and written informed consent was obtained for their participation and use of their data for research and educational purposes. A total of 105 patients aged between 18 and 60 years, classified as American Society of Anesthesiologists Physical Status grades I and II and scheduled to undergo FESS under general anaesthesia, were randomly allocated into three groups using a computer-generated random table. Allocation concealment was achieved using the sequentially numbered and sealed opaque envelope method. Patients allergic to the studied drugs, hypertension, diabetes mellitus, and coagulopathies, those on medications influencing coagulation, coronary artery disease, renal, hepatic, or cerebral insufficiency, and pregnant patients were excluded from the study.

All patients were orally administered 0.25 mg alprazolam and 40 mg pantoprazole before surgery. On the day of surgery, peripheral venous access was secured, and basic standard monitors were used. Premedication on the day of surgery included 0.004 mg kg^-1 ^glycopyrrolate, 2 µg kg^-1^ fentanyl, followed by propofol induction (2 mg kg^-1^) titrated to loss of verbal contact. This was further followed by administration of 0.1 mg kg^-1 ^vecuronium for endotracheal intubation and throat packing. General anaesthesia was maintained using sevoflurane adjusted to 1 minimum alveolar concentration, with maintenance doses of IV vecuronium (0.05 mg kg^-1^) administered if required based on clinical assessment of increased peak airway pressures, spontaneous movements in the reservoir bag, and sudden increases in pulse rate and blood pressure. Patients received mechanical ventilation using the volume-controlled mode with a tidal volume of 6-7 mL kg^-1^ and respiratory rate adjustment to maintain an end-tidal carbon dioxide level of 35-40 mmHg, supplemented with positive end-expiratory pressure set at 5 cmH_2_O using an oxygen/air mixture. Prior to initiating drug infusion, the nasal mucosa of all patients was infiltrated with 5 mL of a solution containing 1 mg adrenaline in 200 mL of normal saline by the surgeon. In the investigation, patients were allocated into three groups: Group magnesium sulfate n = 35 received a loading dose of monosodium glutamate at 25 mg kg^-1^ followed by an infusion of 15 mg kg^-1^ h^-1^; Group propofol n = 35 received a propofol infusion of 10 µg kg^-1^ min^-1^; Group lignocaine n = 35 received a lignocaine infusion of 2 mg kg^-1^ h^-1^. Infusions began after securing the throat pack. Additionally, all patients received an injection of 15 mg kg^-1^ paracetamol. In the event of bradycardia [heart rate (HR) less than 45 bpm], 0.6 mg atropine was intravenously administered. In cases of hypotension [mean arterial pressure (MAP) <60 mmHg], the study drug infusions were stopped, and vasoconstrictors like mephentermine or phenylephrine were administered, along with the titration of the inhalational agent. These patients were subsequently excluded from the study. HR, MAP, systolic blood pressure, and diastolic blood pressure were recorded every 5 min for the first 30 min, followed by every 10 min until the end of the procedure. Drug infusions were discontinued at the end of the procedure, and patients were extubated after the reversal of residual neuromuscular blockade using neostigmine at 0.05 mg kg^-1^ and glycopyrrolate at 0.01 mg/kg based on predefined criteria. The primary outcome was the comparison of the attenuation of hemodynamic responses among the groups. Secondary outcomes included the quality of the surgical field, recovery time, and the total neuromuscular dose requirement during FESS. The attenuation of hemodynamic response was defined as a reduction or moderation of changes in hemodynamics, specifically HR and MAP by 15%. Recovery time was defined as the interval between discontinuation of anesthesia and eye-opening to verbal commands. The surgical field was assessed using the Fromme-Boezaart grading scale, which categorizes the surgical field as follows: 0 = No bleeding; 1 = Slight bleeding, no suctioning of blood required; 2 = Slight bleeding, occasional suctioning required, surgical field not threatened; 3 = Slight bleeding, frequent suctioning required, bleeding threatens the surgical field a few seconds after suction is removed; 4 = Moderate bleeding, frequent suctioning required, bleeding threatens the surgical field immediately after suction is removed; 5 = Severe bleeding, constant suctioning required, bleeding appears faster than can be removed by suction, surgical field severely threatened, and surgery not possible.^5, 6^ A surgical field score of 0-2 was deemed satisfactory. Furthermore, the total dose of muscle relaxant was standardized using vecuronium, administered at an initial loading dose of 0.1 mg kg^-1^, followed by a maintenance dose of 0.05 mg kg^-1^ whenever the patient showed signs of spontaneous effort. The total dose utilized by each patient was recorded.

Sample size calculations were performed using G*Power software. A repeated measures analysis of variance (ANOVA) with a within-between interaction was chosen as the statistical test. The parameters used for the calculation were as follows: effect size (f) = 0.1, significance level (α) = 0.05, desired power (1-β) = 0.80, number of groups = 3, number of measurements within each group = 10, correlation among repeated measures = 0.5, and non-sphericity correction (ε) = 1. The sample size calculation yielded a total sample size of 105.

The data were analyzed using number cruncher statistical systems version 9.0.8 software (Utah, USA). Continuous data were represented as means, ordinal data as medians with interquartile ranges, and categorical data as ratios or percentages. ANOVA was employed to compare continuous data among the three groups and hemodynamic parameters, whereas the chi-square test was performed for categorical data. A significance level of *P* < 0.05 was considered statistically significant.

## Results

**Figure 1:**
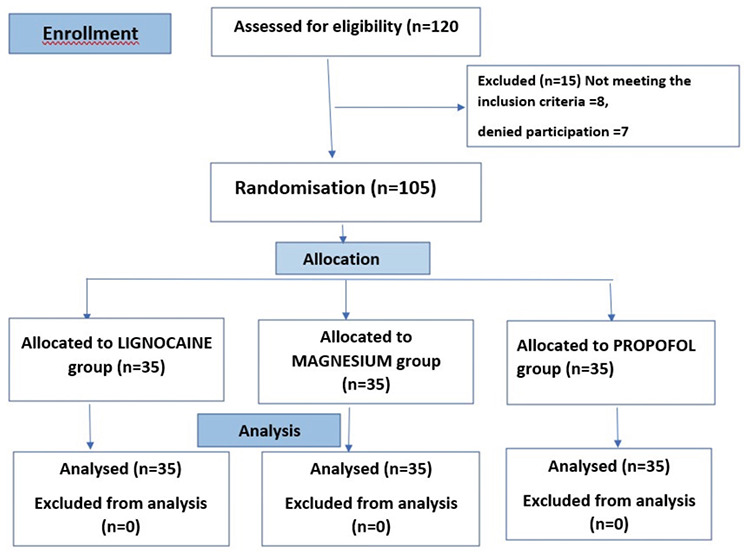
Consolidated standards of reporting trials (CONSORTs) flow of participants.

**Figure 2:**
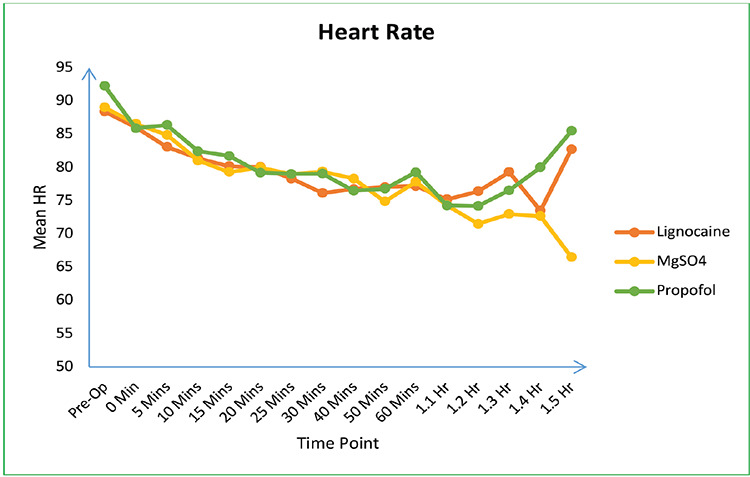
Heart rate variability among the groups.

**Figure 3:**
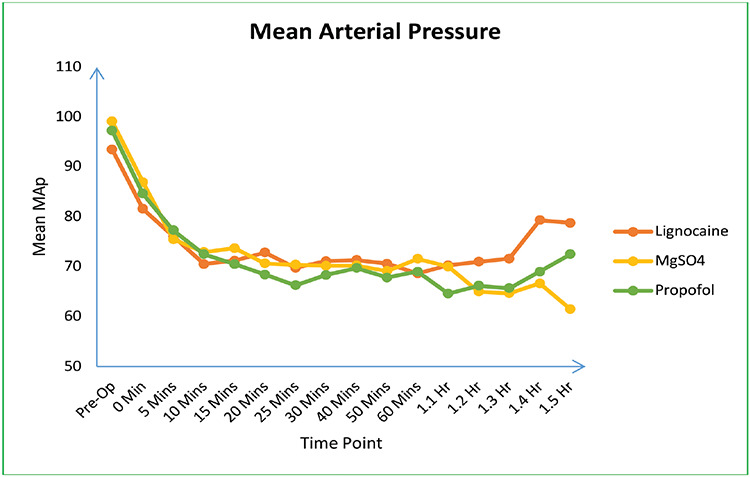
Variation in mean arterial pressure among the groups.

In this study, a total of 120 patients underwent eligibility screening. Of these, 15 were excluded and 105 were enrolled in the trial (Figure 1). All eligible participants were monitored throughout the trial period and were included in the analysis. The three groups were comparable in terms of demographic characteristics, baseline variables, and drug infusion time (Table 1). Each group exhibited a significant decrease in HR and MAP from baseline; however, no statistically significant differences were observed among the groups (Table 2, Figures 2 and 3). A significant difference in the MAP was noted from 1.2 to 1.5 h, whereas the other parameters showed no variation, and no clear rationale was provided for this statistically significant disparity.

Furthermore, the surgical field scores were comparable among the three groups (Table 1). Nonetheless, the recovery time was significantly longer in the magnesium sulfate group [10.94 min (2.45)] than in the lignocaine [4.37 min (1.03)] (95% confidence interval (CI) -7.32, -5.83; *P*=0.000] and propofol groups [4.60 min (0.60)] (95% CI 5.60, 7.095; *P*=0.000). Notably, the difference in recovery time between the lignocaine and propofol groups was not statistically significant (95% CI -0.97, 0.52, *P*=0.545).

The total neuromuscular blocking agent used was significantly lower in the magnesium sulfate group [5.89 mg (0.47)] than in the lignocaine group [6.26 mg (0.56)] (95% CI 0.66, 0.03; *P*=0.035). However, it was comparable to the propofol group [6.20 mg (1.02)] (95% CI 0.29, 0.40; *P*=0.073). Conversely, no significant difference in the total neuromuscular blocking agent dose was observed between the propofol and lignocaine groups (95% CI -0.29, -0.40; *P*=0.743).

## Discussion

FESS is one of the most commonly performed procedures for rhinosinusitis. This technique involves the use of an endoscope and forceps within the nasal cavity, which may lead to bleeding from the highly vascular nasal mucosa. Minimizing this bleeding improves the quality of the surgical field, shortens the operative time, and lowers the risk of major complications.^1, 7, 8^ Attenuating the hemodynamic response is crucial for reducing surgical-site bleeding. This involves reducing blood pressure by 30-40% below the baseline and maintaining this level throughout the surgery while ensuring adequate perfusion to vital organs.^9^ Notably, attenuation of hemodynamic responses can be achieved using a variety of drugs like sodium nitroprusside, nitroglycerin, inhaled anaesthetics, beta-blockers, propofol, dexmedetomidine, lignocaine, and magnesium sulfate.^10^ The ideal agent should be a well-known drug that is easy to use, has rapid onset and remission, and has minimal side effects. In our study, we compared the hypotensive properties of three drugs-propofol, lignocaine, and magnesium sulfate. Although the efficacy of these drugs in blunting hemodynamic responses has been previously investigated, a direct comparison among these drugs has not been conducted.^11, 12, 13^

 We observed a favorable hemodynamic attenuation response with all three drugs, although there was no statistically significant difference among the groups. In a double-blind randomized controlled study, Omar^11^ found that intravenous lignocaine infusion (1.5 mg kg^-1^ h^-1^) resulted in controlled hypotension, stable hemodynamics, and improved surgical conditions at all time points in patients undergoing FESS. Similarly, our study demonstrated stable hemodynamics, controlled hypotension at all time points, and satisfactory surgical field scores with intravenous lignocaine infusion. This hypotensive effect of lignocaine can be attributed to its negative inotropic effect and ability to blunt airway reflexes to the endotracheal tube.^11, 14, 15^ The reductions in MAP and good surgical field scores in the lignocaine group were comparable to those in the magnesium sulfate and propofol groups. Moreover, propofol infusion was equally effective in attenuating hemodynamic response. The proposed mechanisms include vasodilation, a decrease in systemic vascular resistance, and a negative inotropic effect.^16^ In this regard, Gupta et al.^13^ also observed hemodynamic control and satisfactory surgical field scores with propofol during FESS.

Similar to our findings, Elsharnouby and Elsharnouby^17^ observed a significant reduction in MAP and HR with the use of magnesium sulfate for controlled hypotension in patients undergoing FESS. We found that the reductions in MAP and HR in the magnesium sulphate group was comparable to those in the propofol and lignocaine groups. Additionally, we noted a statistically significant decrease in MAP from 1.2 to 1.5 h in the magnesium sulfate group compared with the lignocaine and propofol groups. However, this difference was not clinically significant, and none of the patients in the magnesium sulfate group required vasopressor therapy or discontinuation of drug infusion.

Furthermore, we observed that the recovery time was notably longer when using magnesium sulfate than when using lignocaine and propofol. Chhabra et al.^12^ similarly found an extended recovery time of 10.78 min (3.44) with magnesium sulfate, which was similar to our finding of 10.94 min (2.45) in the same group. In another study, Soliman and Fouad^18^ reported a significantly prolonged extubation time of 13.2 min (1.75) with magnesium sulfate. Additionally, Abu-sinna and Abdelrahman^19^ documented an extended recovery time of 5.2 min (1.8) with propofol infusion in patients undergoing FESS, a finding comparable to our observation of 4.60 min (0.60) with propofol infusion.

Furthermore, the requirement for a total neuromuscular blocking drug was significantly lower with magnesium sulfate than with propofol, although it was similar to lignocaine. The reduced dosage of neuromuscular blocking agents in these patients may be due to the enhancement of nondepolarizing muscle relaxants by magnesium sulfate.^20^

In addition, we noted satisfactory surgical field scores across all three groups, with no significant difference among them. Notably, Elsharnouby and Elsharnouby’s^17^ study demonstrated significantly improved surgical field scores with magnesium sulfate compared with the control group. Similarly, Bharathwaj and Kamath^21^ reported surgical field scores of 2-3 when using propofol in FESS patients, a finding consistent with our own results.

### Study Limitations

The limitations of our study included the lack of comparisons regarding the time required to attain the target MAP and the subjectivity inherent in evaluating the surgical field score. Additionally, train-of-four monitoring was not conducted during the procedures. Double blinding was not feasible because an additional bolus dose was administered alongside the infusion in the magnesium sulfate group, which compromised the blinding process. Furthermore, propofol, with its milky white appearance, was visually detectable within the infusion line.

## Conclusion

Propofol, magnesium sulfate, and lignocaine had comparable efficacy in attenuating hemodynamic response during surgery and achieving a satisfactory surgical field. However, recovery time was notably prolonged with magnesium sulphate compared to propofol and lignocaine. Furthermore, magnesium sulfate resulted in a significantly lower total dose requirement of neuromuscular blocking agents compared with lignocaine.

## Figures and Tables

**Table 1. Demographic Characteristics and Baseline Variables:** 

	**Lignocaine** **Mean (SD)**	**Magnesium sulfate** **Mean (SD)**	**Propofol** **Mean (SD)**	***P* value**
Age (years)	34.11 (8.79)	34.97 (8.24)	37.71 (10.41)	0.123
Gender (Male/Female)	18/17	14/21	19/16	0.449
Weight (kg)	64.69 (6.35)	65.17 (6.10)	64.11 (4.90)	0.749
Baseline HR (min-1)	88.4 (13.079)	89.03 (12.965)	92.26 (14.084)	0.436
Baseline mean arterial pressure (mmHg)	93.51 (16.136)	99.11 (18.149)	97.26 (13.05)	0.329
Drug infusion time (min)	62.57 (22.79)	60.85 (26.71)	61.42 (26.36)	0.959
Recovery time (min)	4.37 (1.03)	10.94 (2.45)	4.60 (0.60)	<0.001
Total NMBA (mg)	6.26 (0.56)	5.89 (0.47)	6.20 (1.02)	0.075
Surgical field score	1.83 (0.62)	1.91 (0.74)	2.06 (0.34)	0.267

Values are expressed as mean (SD) or proportion

SD, standard deviation; HR, heart rate; MAP, mean arterial pressure; NMBA, neuromuscular blocking agent.

**Table 2. Variation of Mean Arterial Pressure and Heart Rate Among the Three Groups:** 

**Time**	**Mean arterial pressure** **Mean (SD) [mmHg]**			***P* value**	**Heart rate** **Mean (SD) [min^−1^]**			***P* value**
	**Lignocaine**	**Magnesium sulfate**	**Propofol**		**Lignocaine**	**Magnesium sulfate**	**Propofol**	
Pre-op	93.51 (16.136)	99.11 (18.14)	97.26 (13.05)	0.329	88.4 (13.079)	89.03 (12.96)	92.26 (14.08)	0.436
0 min	81.6 (14.72)	86.94 (17.57)	84.63 (13.46)	0.348	86.06 (12.286)	86.57 (10.13)	85.91 (13.45)	0.972
5 min	76.09 (12.31)	75.49 (10.38)	77.29 (7.482)	0.757	83.09 (12.356)	84.89 (7.24)	86.37 (12.76)	0.465
10 min	70.54 (9.19)	72.91 (6.90)	72.51 (8.552)	0.442	81.4 (11.413)	81.03 (8.29)	82.43 (10.64)	0.838
15 min	71.2 (7.31)	73.74 (8.84)	70.54 (7.052)	0.196	80.2 (13.807)	79.31 (9.62)	81.74 (9.754)	0.659
20 min	72.83 (7.30)	70.66 (5.567)	68.4 (7.453)	0.029	80.06 (13.104)	80 (8.647)	79.2 (8.881)	0.929
25 min	69.77 (7.04)	70.4 (6.549)	66.26 (4.967)	0.014	78.34 (12.105)	79 (7.742)	79.03 (8.631)	0.945
30 min	71.09 (6.59)	70.17 (6.675)	68.34 (5.861)	0.193	76.14 (12.666)	79.37 (7.923)	79.06 (10.29)	0.367
40 min	71.31 (7.62)	70.23 (6.916)	69.74 (6.771)	0.64	76.74 (12.816)	78.34 (8.349)	76.51 (9.577)	0.727
50 min	70.6 (5.36)	69.13 (7.182)	67.84 (10.82)	0.52	77.08 (14.192)	74.88 (9.695)	76.79 (11.48)	0.843
60 min	68.64 (4.71)	71.57 (7.377)	69 (6.738)	0.354	77.18 (14.789)	77.86 (12.90)	79.27 (15.46)	0.926
1.1 h	70.28 (6.22)	70 (8.571)	64.6 (4.949)	0.091	75.22 (14.926)	74.33 (8.239)	74.3 (8.932)	0.972
1.2 h	71 (7.69)	65 (2)	66.2 (4.756)	0.047	76.4 (15.231)	71.5 (10.268)	74.2 (7.7)	0.66
1.3 h	71.62 (6.09)	64.67 (1.862)	65.67 (3.122)	0.005	79.31 (15.091)	73 (12.992)	76.56 (7.435)	0.601
1.4 h	79.33 (12.67)	66.67 (1.366)	69 (3.098)	0.025	73.5 (17.05)	72.67 (13.09)	80 (4.472)	0.561
1.5 h	78.75 (7.08)	61.5 (9.815)	72.5 (1.732)	0.021	82.75 (24.047)	66.5 (10.97)	85.5 (1.732)	0.219

Values are expressed as mean (SD)

Pre-op, Preoperative; SD, standard deviation.
